# Idiopathic Epilepsy Risk Allele Trends in Belgian Tervuren: A Longitudinal Genetic Analysis

**DOI:** 10.3390/genes15010114

**Published:** 2024-01-18

**Authors:** Nathan Kinsey, Janelle M. Belanger, Paul J. J. Mandigers, Peter A. Leegwater, Tiina Heinonen, Marjo K. Hytönen, Hannes Lohi, Elaine A. Ostrander, Anita M. Oberbauer

**Affiliations:** 1 Department of Animal Science, University of California, Davis, CA 95616, USA; natkinsey@ucdavis.edu (N.K.); jmbelanger@ucdavis.edu (J.M.B.); 2 Department of Clinical Sciences, Utrecht University, Yalelaan 108, 3584 CM Utrecht, The Netherlands; p.j.j.mandigers@uu.nl (P.J.J.M.); p.a.j.leegwater@uu.nl (P.A.L.); 3 Department of Medical and Clinical Genetics, University of Helsinki, 00014 Helsinki, Finland; tiina.heinonen@helsinki.fi (T.H.); marjo.hytonen@helsinki.fi (M.K.H.); hannes.lohi@helsinki.fi (H.L.); 4 Department of Veterinary Biosciences, University of Helsinki, 00014 Helsinki, Finland; 5 Folkhälsan Research Center, 00290 Helsinki, Finland; 6 National Human Genome Research Institute, National Institutes of Health, Bethesda, MD 20892, USA; eostrand@mail.nih.gov

**Keywords:** idiopathic epilepsy, seizure, Belgian Tervuren, selective breeding, neurological disorder, dog

## Abstract

Idiopathic epilepsy (IE) has been known to be inherited in the Belgian Tervuren for many decades. Risk genotypes for IE in this breed have recently been identified on Canis familiaris chromosomes (CFA) 14 and 37. In the current study, the allele frequencies of these loci were analyzed to determine whether dog breeders had employed a purposeful selection against IE, leading to a reduction in risk-associated allele frequency within the breed over time. The allele frequencies of two generational groupings of Belgian Tervuren with and without IE were compared. Allele frequencies for risk-associated alleles on CFA14 were unchanged between 1985 and 2015, whereas those on CFA37 increased during that time in the control population (*p* < 0.05). In contrast, dogs with IE showed a decrease (*p* < 0.05) in the IE risk-associated allele frequency at the CFA37 locus. Seizure prevalence in the Belgian Tervuren appears to be increasing. These results suggest that, despite awareness that IE is inherited, selection against IE has not been successful.

## 1. Introduction

Idiopathic epilepsy (IE) is a neurological disorder characterized by repeated seizure activity [1,2,3,4]. Idiopathic epilepsy is distinct from other forms of epilepsy in that it does not stem from a known underlying cause aside from the presumed contribution of genetics [5]. Epilepsy is one of the most common neurological diseases in dogs [6,7,8,9], with some breeds, including both the Belgian Sheepdog and Belgian Tervuren, having a combined prevalence estimate of 9.5% [10,11], and Belgian Tervuren alone has a reported prevalence of 17% [12]. This disease represents a pressing issue for dog owners [13,14,15] and animal welfare [16,17,18,19,20,21], as seizing episodes are acutely stressful for the affected dog. Additionally, various clinical tests must be administered to confidently diagnose IE, which is time-consuming and expensive [22,23]. In some cases, the frequency and severity of seizures rise dramatically, forcing owners to elect for euthanasia [24,25,26,27]. 

It has been well known for several decades that IE in dogs is inherited [28,29,30,31,32]. Additionally, modeling selection against IE in the Belgian Tervuren indicates that purposeful selective breeding could prove fruitful in reducing disease incidence [33]. Research to identify these genetic contributions has included multiple genome-wide association studies conducted in the Belgian Sheepdog and Tervuren; those studies identified regions associated with IE risk on Canis familiaris chromosomes (CFA) 14 and 37 [34,35]. Four single-nucleotide polymorphisms (SNPs) on CFA14 form a risk haplotype near the *RAPGEF5* gene, which has a presumed role in neurology [36], and its downregulation is associated with focal seizures [37]. Two risk-associated SNPs on CFA37 are near *ADAM23* and *KLF7,* genes related to epilepsy in dogs [38,39] and modulating neuronal morphogenesis [40,41,42,43], respectively.

This study evaluated the IE-associated risk alleles and interacting haplotypes on CFA14 and 37 to determine if there was a reduction in allele frequency over a thirty-year period in Belgian Tervuren due to heightened awareness of the disease and phenotypic selection against it. We aimed to determine if the allele and/or haplotype frequency changed over time and, if so, the magnitude of this response to selection. Additionally, we sought to determine an estimate of disease prevalence in recent years to confirm if IE has become less common over time.

## 2. Materials and Methods

Genotypes for the CFA14 and 37 risk loci were determined for 203 Belgian Tervuren (122 collected in the United States, 48 in Finland, and 33 in The Netherlands), as previously described [44]. Samples of buccal swabs or whole blood were submitted by owners and veterinarians. Dogs were genotyped with the Illumina CanineHD BeadChip (San Diego, CA, USA), with 173,662 SNPs based on the CanFam3.1 reference genome [45] or via direct PCR and Sanger sequencing of the CFA14 and CFA37 SNPs [44]. To account for relatedness, a subset of 175 unrelated dogs with parent–offspring pairs and full siblings excluded was also evaluated. Data processing was performed using an R script (Script S1) built using the Tidyverse framework and written in RStudio 4.3.2 [46,47]. Dogs were classified as healthy controls or having IE based on the criteria previously described [35]. Specifically, healthy controls were dogs over the age of 7 years (mode 10 years) that lacked any reported health issues. Dogs with IE were classified using the Tier I confidence level diagnostic criteria set by the International Veterinary Epilepsy Task Force [5]. These criteria for IE diagnosis are based on the exclusion of other seizure disorders; if all examinations are conducted without identifying a clear cause of seizure activity, IE is diagnosed. Following these guidelines, IE dogs had two or more generalized seizures at least 24 h apart, were over the age of 1 year at the time of disease onset (mode 3 years), and were less than 6 years of age. Additionally, IE dogs had unremarkable physical and neurological examinations as determined by a veterinarian, meaning that their seizures had no suspected cause aside from a presumed genetic contribution.

The A allele of the BICF2S23230472 SNP was representative of the four-base risk haplotype on CFA14, and the G allele of the BICF2P271491 SNP was representative of the two-base risk haplotype on CFA37. The haplotype blocks on each chromosome have been previously shown to have strong linkage disequilibrium (D’ = 1 and *r*^2^ = 0.96–1), allowing for any one of these SNPs to act as a reliable proxy for the co-segregating block [35]. The data were divided by date of birth, with dogs born between 1985 and 1999 categorized as the older group and dogs born between 2000 and 2015 categorized as the recent group. Dogs were genotyped using the same platforms, irrespective of their age group. It is known that population substructure in dogs can vary by geographical region [48], which could potentially skew the results if the age groups were not similarly distributed between sampled populations. To determine the balance of age distribution across the three regions where samples were collected, a value of Cramer’s V was calculated for the number of dogs in each age group using a 2 × 3 contingency table on the VassarStats web tool [49,50]. This is a measure ranging from 0 (complete independence between the variables and assayed groups) to 1 (a strong association) [49]. Alleles were counted for the CFA14 and CFA37 SNPs in unrelated and related datasets, divided by age group (Script S1). Fisher exact tests were performed on allele counts to detect statistically significant changes over time using a 2 × 2 contingency table on the VassarStats web tool 8.6 [50,51]. Combined risk haplotypes were assessed in the unrelated cohort by grouping haplotypes into high or low IE risk, as previously modeled via logistic regression [35]. Fisher exact tests were performed on haplotype counts to detect statistically significant changes over time, using the same procedure as the allele counts [50,51].

Complete and updated health information and genetic samples for Belgian Tervuren from around the world have been collected over the past 30 years in our laboratory for various genomic diseases and behavioral work. From these data, a cohort of 144 Belgian Tervuren (n = 39 IE, n = 105 healthy), using the same health criteria as previously described [12], born between 2000 and 2020 were used to calculate current IE prevalence.

## 3. Results

Only a moderate deviation was detected (Cramer’s V = 0.291) between geographical regions, indicating acceptable age group homogeneity between the sampled populations. There were no statistically significant changes in the CFA14 risk haplotype allele frequency over time (Table 1). The CFA37 locus, however, showed an increase in the IE risk-associated G allele frequency over time in the overall population (*p* < 0.09). This increase was even more pronounced (*p* < 0.05) in the control dogs from this population, whereas dogs with IE showed a decrease (*p* < 0.05) in the G risk-associated allele. The unrelated Belgian Tervuren revealed the same trends; the CFA14 haplotype remained unchanged through time, whereas the CFA37 risk-associated allele frequency rose in the controls and in the total population, although failing to reach significance, and decreased in the IE dogs (*p* < 0.05). 

**Figure 1:**
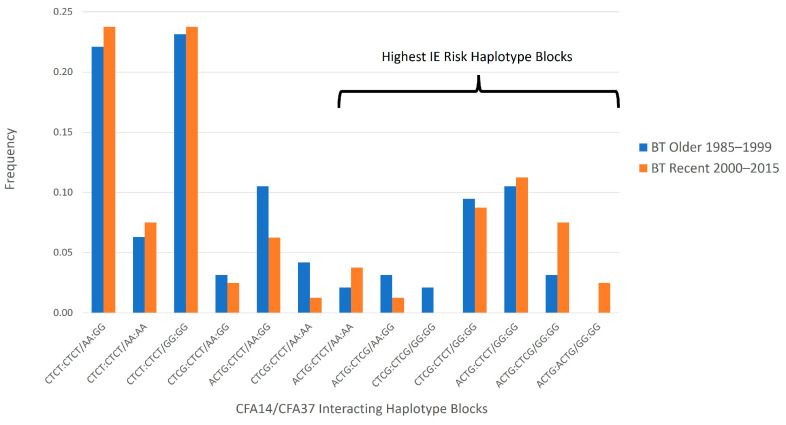
Changes in observed CFA14 and CFA37 interacting risk haplotype block frequency over time in 175 unrelated Belgian Tervuren (BT). Haplotypes are ordered by increasing risk, with the haplotype interactions on the far right being highly associated with IE in Belgian Tervuren [35].

When the combined CFA14 and CFA37 risk haplotype blocks were assessed (Figure 1), there were no statistically significant changes in haplotype block frequency over time in the unrelated Belgian Tervuren population (*p* > 0.1). Similarly, when the unrelated population was divided by IE status, neither the epileptic nor control dogs demonstrated a statistically significant change in haplotype frequency over time (*p* > 0.1).

In the cohort of 144 Belgian Tervuren born between 2000 and 2020, 39 were reported to have seized more than once (27.1% prevalence), and 105 were over the age of 7 years and reported to be healthy and free from any underlying health conditions (72.9%).

## 4. Discussion

With increased awareness of IE in Belgian Tervuren [52], we anticipated that the prevalence of IE risk-associated alleles and haplotype frequencies would decrease over time. That was not the case; the risk-associated allele frequencies were either unchanged (CFA14) or increased (CFA37). This could represent a lack of purposeful selection against IE in the breed or difficulty using phenotypic selection to affect changes at the two loci. However, the CFA37 locus, associated with IE risk in many different dog breeds [53], demonstrated a significant decrease in the IE dogs over time, perhaps reflecting the complex nature of the genetic contributors of IE and the rise of additional risk-associated loci over time. Similarly, haplotypes that have been previously associated with IE also failed to demonstrate reduced frequency in the population. This further supports the possibility that dog breeders may not have been successful in breeding away from the disease. These data suggest that IE prevalence has increased over time. The prevalence of IE in Belgian Tervuren in the 1980s, derived from the American Belgian Tervuren Club Survey, was 17% [12], whereas the current prevalence is just over 27% when using the same criteria to define IE. The rise in risk allele frequencies and population prevalence suggests that attempts to breed away from IE have not been implemented or successful at the population level.

While IE is a genetic disease, it is important to consider that the seizures themselves can be triggered by environmental factors such as stress, excitement, novel stimuli, and extreme weather conditions [54]. Future research may benefit from considering how the environment may have changed over time, affecting the observed IE in the Belgian Tervuren. For example, preliminary research suggests that global climate change may both trigger seizures and exacerbate other triggers, such as disruptions in sleep cycles or elevated stress [55]. Mitigating such comorbidities may be vital in addition to the existing IE care regimen prescribed for a given dog [56]. These environmental factors may interact with the genetic factors, or perhaps even the medication used to treat the disease and contribute to the observed IE [57].

A decrease in CFA37 risk-associated allele frequency in the IE dogs might suggest that the driving genetic factors of IE in Belgian Tervuren have become more heterogeneous over time, highlighting the need for continued research into the disease’s etiology. This also may explain the seemingly disparate trends of decreasing risk allele frequency and increasing IE prevalence; if the common genetic causes of IE become more varied than the previously described high-impact loci, the response to phenotypic selection against the disease may increasingly be diminished in the face of increased heterogeneity. While using genetic tests for risk alleles may prove invaluable for disease management [58], the present study suggests that such selection will only be effective if loci beyond the CFA14 and CFA37 haplotypes are discovered and their interactions investigated thoroughly. Further investigation into the genetic basis of IE in Belgian Tervuren is crucial to improve the welfare of the breed and to give owners a tool to guide breeding decisions in the future.

## Figures and Tables

**Table 1:** Changes in idiopathic epilepsy (IE) risk-associated allele frequency over time in dogs born between 1985 and1999 (older cohort) compared to dogs born between 2000 and 2015 (recent cohort) at both Canis familiaris chromosome (CFA) 14 and 37 loci.

	CFA14—BICF2S23230472			CFA37—BICF2P271491		
	Risk-Allele A Frequency Older Cohort	Risk-Allele A Frequency Recent Cohort	*p*-Value	Risk-Allele G Frequency Older Cohort	Risk-Allele G Frequency Recent Cohort	*p*-Value
All dogs						
Control	0.14 (*n* = 85)	0.15 (*n* = 66)	0.41	0.62 (*n* = 85)	0.73 (*n* = 66)	0.03
IE	0.25 (*n* = 16)	0.21 (*n* = 36)	0.41	0.91 (*n* = 16)	0.74 (*n* = 36)	0.04
Total	0.15 (*n* = 101)	0.17 (*n* = 102)	0.36	0.67 (*n* = 101)	0.74 (*n* = 102)	0.09
Unrelated dogs						
Control	0.13 (*n* = 80)	0.14 (*n* = 52)	0.45	0.64 (*n* = 80)	0.72 (*n* = 52)	0.10
IE	0.23 (*n* = 15)	0.23 (*n* = 28)	0.60	0.90 (*n* = 15)	0.68 (*n* = 28)	0.02
Total	0.15 (*n* = 95)	0.18 (*n* = 80)	0.29	0.68 (*n* = 95)	0.71 (*n* = 80)	0.33

## Data Availability

The data presented in this study are openly available in a previously published *Genes* communication [44].

## Supplementary Materials

The following supporting information can be downloaded at: https://www.mdpi.com/article/10.3390/genes15010114/s1, Script S1: R script for identifying related dogs and counting alleles.
